# The Detection and Verification of Two Heterogeneous Subgroups and a Risk Model Based on Ferroptosis-Related Genes in Hepatocellular Carcinoma

**DOI:** 10.1155/2022/1182383

**Published:** 2022-03-12

**Authors:** Jiang Li, Haisu Tao, Wenqiang Wang, Jian Li, Erlei Zhang

**Affiliations:** ^1^Hepatic Surgery Center, Tongji Hospital, Tongji Medical College, Huazhong University of Science and Technology, No. 1095, Jiefang Avenue, Wuhan 430030, Hubei, China; ^2^Department of Hepatobiliary Surgery, College of Medicine, First Affiliated Hospital of Shihezi University Medical College, North 2nd Road, Shihezi, Xinjiang Uygur Autonomous Region, China

## Abstract

^**#**^ *Background*. Because of the heterogeneity of hepatocellular carcinoma (HCC) and the complex nature of the tumor microenvironment (TME), the long-term efficacy of therapy continues to be a clinical challenge. It is necessary to classify and refine the appropriate treatment intervention decision-making in this kind of tumor. *Methods*. We used “ConsensusClusterPlus” to establish a stable molecular classification based on the ferroptosis-related genes (FRGs) expression obtained from FerrDb. The clinical features, immune infiltration, DNA damage, and genomic changes of different subclasses were evaluated. The least absolute shrinkage and selection operator regression (LASSO) method and univariate Cox regression were utilized to construct the ferroptosis-related prognosis risk score (FPRS) model, and the association between the FPRS model and HCC molecular characteristics, immune features, and immunotherapy was studied. *Results*. We identified two ferroptosis subclasses, C1 with poor prognosis and a higher proportion of patients in the middle and late stages infected with HBV and HCV, having higher DNA damage including aneuploidy, HRD, fraction altered, and the number of segments, and higher probability of gene mutation and copy number mutation. FPRS model was constructed on the basis of differentially expressed genes (DEGs) between C1 and C2, which showed a higher area under the curve (AUC) in predicting overall survival rate in the training set and independent verification cohort and could reflect the clinical characteristics and response to immunotherapy of different patients, being an independent prognostic factor of HCC. *Conclusion*. Here, we revealed two novel molecular subgroups based on FRGs and develop an FPRS model consisting of six genes that can help predict prognosis and select patients suitable for immunotherapy.

## 1. Introduction

Primary liver cancer has been reported to be the fifth-highest occurring incidence of cancer in the world, which comprises hepatocellular carcinoma (HCC) (accounting for approximately 75%–85% of all incidents) and intrahepatic cholangiocarcinoma (accounting for approximately 10%–15% of all incidents) and other rare types [1]. As the most prevalent type of primary liver cancer, the treatment of HCC has been restricted by tumor heterogeneity, which greatly limits the progress of individualized therapy [2]. The histological definition of morphological heterogeneity of liver cancer has been modified and refined in the medical community to help clinically choose treatment interventions for patients, but this still does not solve all the problems [3]. Precision medicine has been suggested to add a new perspective to individualized cancer diagnosis and targeted therapy by taking into account the heterogeneity of individual patients [4]. Precision medicine focuses on the importance of accurately classifying heterogeneous diseases into more accurate subsets with the aid of powerful identification techniques and the incorporation of clinical characteristics. Furthermore, clinicians should come up with more specific diagnostic and therapeutic approaches for the disease subtype in order to optimize the efficacy and ultimately reduce side effects [5].

Iron toxicity is an iron-dependent cell death program, whose primary feature is the accumulation of lethal amounts of lipid-reactive oxygen species in cells [6]. Over the past few years, studies have suggested that the liver is prone to oxidative damage and iron overload is the cause of liver injury as well as the progression of disease in most liver diseases [7]. Therefore, ferroptosis has attracted wide attention in a variety of liver diseases, including HCC, hepatic fibrosis, liver failure, hepatic ischemia-reperfusion injury, and nonalcoholic steatosis [8]. In hepatocyte-specific Trf knockout mice, feeding a diet with high iron increased their vulnerability to liver fibrosis induced by iron death. And ferroptosis suppressants can restore this condition [9]. A study conducted in mice showed that ferroptosis is an inducer of nonalcoholic steatohepatitis, leading to liver injury, immune cell infiltration, and inflammatory response [10]. Ferroptosis also mediates acetaminophen-induced acute liver failure [11]. Multiple studies pointed to the induction of ferroptosis as a possible effective tumor suppressor mechanism and useful for prognosis prediction in HCC [7]. The late first-line therapeutic drug of HCC, sorafenib, has been proved to be a strong inducer of ferroptosis [12]. Sorafenib increased the survival rate of HCC patients to a certain degree, but it may lead to serious harmful impacts and growing resistance characteristics, resulting in a dismal prognosis [13]. Therefore, it is necessary to identify new molecular markers of ferroptosis and downstream signaling pathways, which will aid in the comprehension of the regulatory mechanism of ferroptosis in the physiopathology of HCC.

At present, there are several systems biology methods to identify biomarkers related to the prognosis of HCC and construct gene features. Liang et al. identified a 10-gene signature in the expression profile of iron death related genes by LASSO regression analysis [14]. Liu et al. analyzed m6A methylation related genes and identified five gene markers with poor prognosis [15]. Xu et al. identified 6-gene signature by Cox regression analysis [16]. All three groups of authors tested their gene signature in the internal data set but did not verify the external independent data set, which means that identifying robust lncRNA signature is still a challenge and more queues are needed to verify the signature.

In this research, we collected samples from four databases, identified two distinct ferroptosis-related subclasses in HCC patients based on the expression of 111 FRGs obtained from the FerrDb website, and discussed the clinical, mutation spectrum, and tumor immunological characteristics between ferroptosis subgroups. In addition, the FPRS model was constructed to quantify the survival probability of HCC patients and to predict the response to immunotherapy. Collectively, this FPRS model may be an excellent predictor of HCC and may give insight into the development of innovative possible therapeutic techniques.

## 2. Materials and Methods

### 2.1. Acquiring and Preprocessing Sample Data

RNA-Seq data containing 365 samples and valid clinical follow-up information were acquired from TCGA-LIHC (https://portal.gdc.cancer.gov/). In addition, transcriptome data and survival messages from 221 cases of GSE14520 [17] and 115 cases of GSE76427 [18] cohorts were collected from the Gene Expression Omnibus (GEO, https://www.ncbi.nlm.nih.gov/geo/). Similarly, the ICGC-LIRI-JP data set in the HCCDB database was also used for the collection of HCC data, including 212 samples. TCGA-LIHC served as the training set, while the other cohort served as the independent verification set. The whole work flow chart of this study is shown in Figure S1.

### 2.2. Collection and Unsupervised Clustering of Ferroptosis-Related Genes (FRGs)

FerrDb (http://www.zhounan.org/ferrdb) is reported to be the first repository of ferroptosis modulators and indicators, as well as ferroptosis-disease connections, which was manually collated [19]. We got 111 FRGs from this website. Then, the FRGs significantly correlated with the prognosis of HCC patients were selected utilizing univariate Cox analysis. According to the levels of FRGs expression, which is significantly correlated with the prognosis of HCC, the R packet ConsensusClusterPlus [20] was used to classify 365 HCC samples from TCGA-LIHC. And the analysis measured the distance by “Euclidean” and performed 500 times resampling iteration for both algorithms with 80% of probe sets being subsampled to ensure the stability of the clustering.

### 2.3. Computation of Molecular Features and Immune Cellular Fraction between Subtypes

Genomic Data Commons Data Portal provided somatic mutation profiles identified by VarScan, which were accessible to download [21]. Somatic mutation frequency of more than 5 percent was regarded to be appropriate for comparing values across different subtypes [22]. The “maftools” package [23] of R software was employed to display the mutation spectrum of each subtype. The relative abundance of 22 different immune cells in distinct subgroups in two HCC cohorts was calculated by executing the CIBERSORT algorithm [24]. The stromal, immune, and ESTIMATE scores of each sample were evaluated by ESTIMATE [25] to determine the degree of immune cell infiltration of each subtype.

### 2.4. Differential Expression Analysis between Molecular Subclasses

The Limma package was employed to identify differentially expressed genes (DEGs) between distinct subgroups in the TCGA-LIHC data set [26]. The genes having an absolute log2 fold change (|logFC|) > 1.0, false discovery rate (FDR) < 0.05, and *P*value <0.01 were defined as DEGs. The “clusterProfiler” package of *R* [27] was applied to implement the Gene Ontology (GO) and Kyoto Encyclopedia of Genes and Genomes (KEGG) pathways analysis on DEGs between distinct subtypes and the critical value was adjusted as *P* < 0.05.

### 2.5. Establishment and Evaluation of Ferroptosis-Related Prognosis Risk Score (FPRS) System

Univariate Cox regression analysis and the least absolute shrinkage and selection operator (Lasso) Cox regression analysis were applied to build the prognostic risk model based on DEGs between distinct subtypes, which was performed using R packet (http://www.rstudio.org) “glmnet.” The specific formula was as follows: HPRS = Σ*βi* × *Expi*, where *β* is the Cox regression coefficient of the corresponding gene, *i* refers to the prognostic related FRGs, and Exp is the prognostic FRGs expression level. Similarly, the accuracy of FPRS model was verified in two independent validation sets. The cut-off point of FPRS in each cohort was obtained according to R packet “survminer.” Patients who were larger than the threshold value were categorized into a high-risk group, and those less than the threshold value were categorized into a low-risk group. The Kaplan–Meier curve was used to display the overall survival (OS) of the sample, and the logarithmic rank test was utilized to determine the statistical difference. The “timeROC” package of R was applied for the generation of receiver operating characteristic (ROC) curve, and the prediction accuracy of the model was examined by calculating the area under the curve (AUC) of one-, three-, and five-year OS.

### 2.6. The Function of Different FPRS Was Analyzed by Gene Set Enrichment Analysis (GSEA)

HALLMARK GSEA was performed to estimate the biological signaling pathways in different risk groups [28]. And single-sample GSEA (ssGSEA) was conducted in the TCGA-LIHC cohort utilizing the “GSVA” package of R to study molecular differences between samples with different FPRS.

### 2.7. Genomic Correlations with the FPRS

Aneuploidy scores, homologous recombination deficiency (HRD), fraction altered, number of segments, and tumor mutation were derived [29]. The differences in these five indicators between the 2 risk groups were examined by Wilcoxon test. The correlation between FPRS and the above five genomic variables was evaluated by Pearson's correlation analysis.

### 2.8. Prediction of Response to Different Treatments

Immune checkpoint expression data were obtained from the HisgAtlas database [30] and compared between TCGA-LIHC risk groups. Immunophenoscore (IPS) can be computed in an unbiased way utilizing machine learning algorithms on the basis of 4 primary gene types (immunomodulators, MHC molecules, effector cells, and immunosuppressive cells) that influence immunogenicity [31]. We acquired the IPS of HCC from the TCIA database (https://tcia.at/home) [32] and compared the IPS of the distinct FPRS risk group in TCGA-LIHC to evaluate the responsiveness to immune checkpoint blocking therapy. The Tumor Immune Dysfunction and Exclusion (TIDE, http://tide.dfci.harvard.edu/) algorithm was run in three cohorts to identify the TIDE score difference between the low- and high-risk groups. We employed the pRRophetic algorithm to estimate the response to sorafenib, docetaxel, paclitaxel, and cisplatin identified by the half-maximal inhibitory concentration (IC50) for each TCGA-LIHC sample on the Genomics of Drug Sensitivity in Cancer (GDSC) database.

### 2.9. Statistical Analysis

All statistical analyses and data visualization were conducted in R (https://www.r-project.org/, version 3.6.3). And all calculated *P* values were two-tailed; *P* < 0.05 was considered significant.

## 3. Results

### 3.1. Two Ferroptosis Clusters in HCC Were Identified by Consensus Clustering Based on FRGs

**Figure 1:**
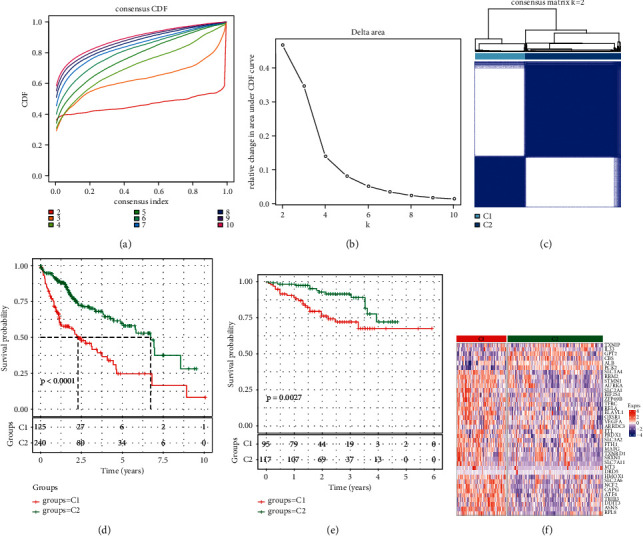
Consensus clustering analysis based on the prognosis on FRGs in HCC. (a) The cumulative distribution function (CDF) of distinct clustering methods from *k* = 2 to 9. (b) The relative alterations of the area under CDF curves with the index from 2 to 9. (c) Clustering heat map of TCGA-LIHC samples with the index *k* = 2. (d) Kaplan–Meier curves for ferroptosis clusters prognosis in TCGA-LIHC cohort. (e) Kaplan–Meier curve of OS between two ferroptosis clusters. (f) The expression heat map of 38 prognostic FRGs in two ferroptosis clusters.

Univariate Cox regression analysis of 111 FRGs selected from FerrDb showed that 38 FRGs were considerably correlated with the prognosis of HCC patients. According to the expression level of these 38 FRGs (Supplementary Table 1), 365 samples in TCGA-LIHC were clustered (Supplementary Table 2). The cumulative distribution function (CDF) of distinct clustering techniques from *k* = 2 to 9 and the relative variations of the area under CDF curves demonstrated that the area under the CDF chart tended to be stable when *k* = 2 (Figures 1(a) and1(b)). Therefore, HCC was divided into two ferroptosis clusters, namely, C1 and C2 (Figure 1(c)). In the TCGA-LIHC cohort, an obvious difference in prognosis between the two ferroptosis clusters was shown, and the prognosis of C2 was significantly stronger than that of C1 (Figure 1(d)). Survival analysis in ICGC yielded the same results (Figure 1(e)). Heat maps of the expression of 38 prognostic FRGs in two ferroptosis clusters showed that most prognostic FRGs were overexpressed in C1 (Figure 1(f)).

### 3.2. Association of Ferroptosis Clusters with Clinical Features

**Figure 2:**
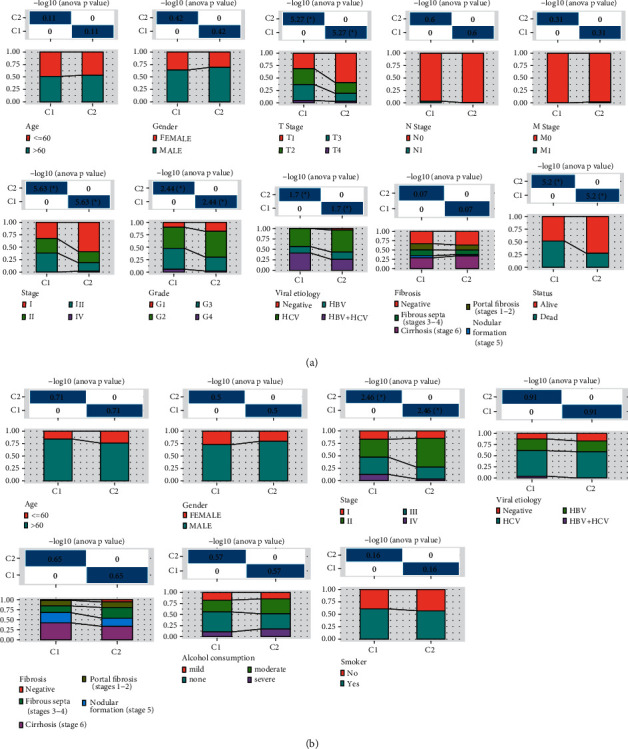
Correlation of ferroptosis clusters with clinical features. (a) The clinicopathological distribution diagram of two ferroptosis clusters in the TCGA-LIHC cohort, including grade, M stage, sex, N stage, T stage, AJCC, age, viral etiology, fibrosis, and life status. (b) In the ICGC cohort, the age, gender, AJCC stage, viral etiology, fibrosis, alcohol consumptions, and smoking proportion distribution differences between C1 and C2; chi-square test; ^*∗*^*P* < 0.05.

Next, the relationship between two ferroptosis clusters and clinicopathological factors was studied. The proportional distribution maps of different clinical bed characteristics are generated. In the TCGA-LIHC cohort, the two ferroptosis clusters did not exhibit any obvious differences in age (age ≤60 and age >60), gender (female and male), life status (alive and dead), M stage (M0 and M1), N stage (N0 and N1), and fibrosis (negative, portal fibrosis, fibrous septa, nodular formation, and cirrhosis) distribution. And the distributions of grade (G1, G2, G3, and G4), AJCC stage (stage I, stage II, stage III, and stage IV), and T stage (T1, T2, T3, and T4), viral etiology (negative, HBV, HCV, and HBV+HCV), and life state (alive and dead) between C1 and C2 in the TCGA-LIHC cohort were significantly different. Among them, C2 samples were often from the AJCC stage, M stage, N stage, T stage, survival patients with low tumor grade and hepatitis C virus (HCV), and hepatitis B virus (HBV) infection (Figure 2(a)). In the ICGC cohort, a significant difference was shown between C1 and C2 only in the proportion of different AJCC stages. In the C1 subtype, stage II and stage III occupy the absolute majority of this subtype in a nearly equal proportion. However, more than half of the samples of the C2 subtype were in stage III. No significant differences were identified in age, gender, viral etiology, fibrosis, and alcohol consumptions, and smoking between the two subtypes in this cohort (Figure 2(b)).

### 3.3. Comparisons of the Somatic Variation between Two Ferroptosis Clusters

**Figure 3:**
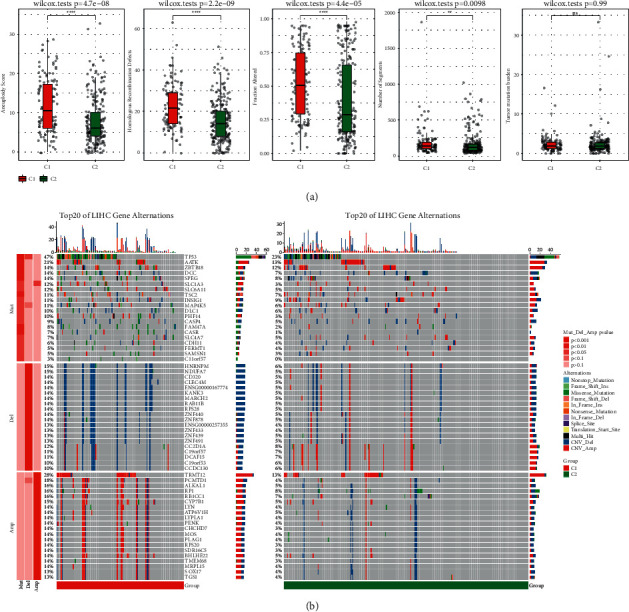
Difference of the somatic variation between two ferroptosis clusters. (a) Relation of DNA damage with ferroptosis subgroups in TCGA-LIHC cohort, including aneuploidy score, HRD, fraction altered, number of segments, and tumor mutation burden; Wilcoxon test. (b) OncoPrint of gene mutation and CNV distribution between C1 and C2 patients. Fisher's test, ^*∗∗*^*P* < 0.01, and ^*∗∗∗∗*^*P* < 0.0001.

To further investigate the molecular mechanism behind the classification of ferroptosis subtypes, mutation spectra of two ferroptosis subtypes were analyzed. The ferroptosis subtypes were associated with measures of DNA damage, including aneuploidy, HRD, fraction altered, and the number of segments. Compared with C1, C2 had a lower aneuploidy score, HRD, fraction altered, and the number of segments. Nevertheless, no significant differences were identified in tumor mutation burden (TMB) between C1 and C2 (Figure 3(a)). OncoPrint of gene mutation distribution between C1 and C2 patients showed a significant association between the ferroptosis subtype and somatic mutations. The relative frequency of 20 altered genes in C1 was high. In addition, in terms of copy number variation (CNV), C1 had a higher frequency of copy number amplification and deletion than C2 (Figure 3(b)).

### 3.4. Differences in Immune-Related Characteristics of Ferroptosis Subtypes

**Figure 4:**
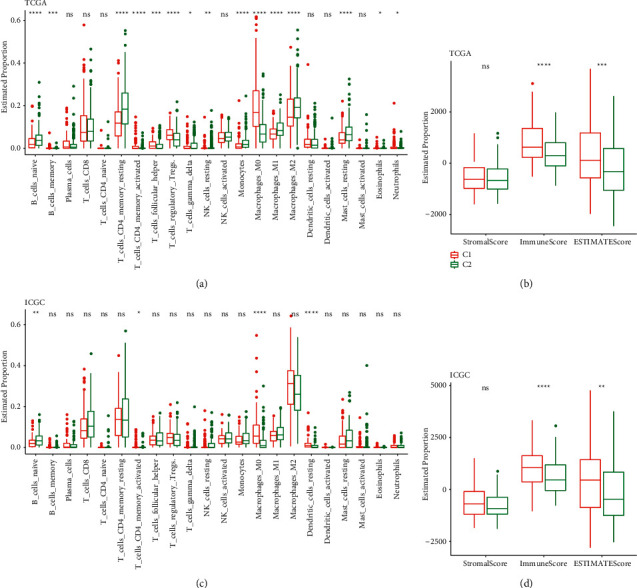
Immune-related features in each ferroptosis subtype. (a) Each immune infiltrating cell abundance of the two ferroptosis subtypes in the TCGA-LIHC cohort. (b) Differences in stromal, immune, and ESTIMATE scores between the two ferroptosis subtypes in the TCGA-LIHC cohort. (c) The abundance of 22 immune infiltrating cells per ferroptosis subtypes in the ICGC cohort. (d) Stromal, immune, and ESTIMATE scores of each ferroptosis subtype in ICGC cohort. ^*∗*^*P* < 0.05; ^*∗∗*^*P* < 0.01; ^*∗∗∗*^*P* < 0.001; ^*∗∗∗∗*^*P* < 0.0001.

To examine the immune heterogeneity between two ferroptosis subtypes, the immune characteristics of two ferroptosis subtypes were analyzed. The abundance of 22 different kinds of immune cells in TCGA-LIHC and ICGC cohort was computed utilizing the CIBERSORT and compared between groups of ferroptosis subtypes. In the TCGA-LIHC cohort, M0 macrophages, regulatory T cells, helper follicular T cells, and activated memory CD4 T cells were strongly enriched in C1, while the cells significantly enriched in C2 included monocytes, resting memory CD4 T cells, naive B cells, M1and M2 macrophages, and resting mast cells (Figure 4(a)). In the ICGC cohort, activated memory CD4 T cells and M0 macrophages, naive B cells, and resting dendritic cells have significantly different abundances in C1 and C2 (Figure 4(c)). By comprehensive analysis of stromal, immune, and ESTIMATE scores of two ferroptosis subtypes in each cohort, C1 was greatly elevated as opposed to C2 (Figures 4(b) and 4(d)).

### 3.5. Identification of Genes Associated with Ferroptosis Phenotype

**Figure 5:**
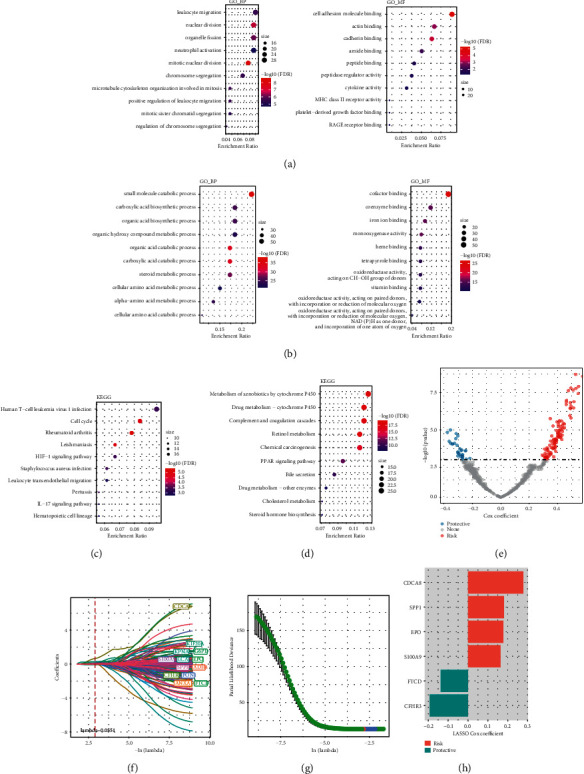
Recognition of genes associated with ferroptosis phenotype. (a) Top 10 GO terms of DEGs between two ferroptosis subtypes in TCGA-LIHC. (b) The KEGG pathways of DEGs between two ferroptosis subtypes in TCGA-LIHC. (c) All DEGs-enriched top 10 GO terms in ICGC cohort. (d) All DEGs-enriched top 10 KEGG pathways in ICGC cohort. (e) Univariate regression between DEGs and HCC prognosis. (f) Distribution of LASSO coefficients of 137 genes with prognostic value. (g) 5-time cross-validation was used to select the best parameters in the model. (h) The coefficient of each gene in the optimal model.

To identify ferroptosis phenotypes related genes, the differential expression analysis of two ferroptosis subtypes was carried out (FDR <0.05 and | log2FC | > log2 (2)), and 324 upregulated differentially expressed genes (DEGs) and 274 downregulated DEGs were identified for the first time. Among them, the top 5 genes with the highest expression in C1 are SPP1, AFP, PKM, CD24, and MYBL2, and the top 5 genes with the highest expression in C2 are TAT, CYP2A6, SLC10A1, CYP3A4, and HPD. The functional enrichment analysis of the DEGs between the two ferroptosis subtypes was carried out, respectively. In TCGA-LIHC, the top GO terms of DEGs included cell division, immune cell activation, cell migration, and cytokine activity (Figure 5(a)). Moreover, all the pathways generated from KEGG analysis were associated with immune responses (Figure 5(c)). For the ICGC cohort, all DEGs-enriched GO terms and KEGG pathways were correlated with the anabolism of cancer cells (Figures 5(b) and 5(d)). Univariate Cox regression analysis illustrated that 137 genes had prognostic significance in 598 DEGs (Figure 5(e)), which were included in LASSO analysis. The best parameter based on 5-time cross-validation was 13 (Figures 5(f) and 5(g)). The stepAIC in the MASS package reduced the number of genes from 13 to 6 and calculated each gene's risk value in the optimal model as shown in Figure 5(h).

### 3.6. Generation and Validation of a Risk Scoring Model Based on Six FRGs

**Figure 6:**
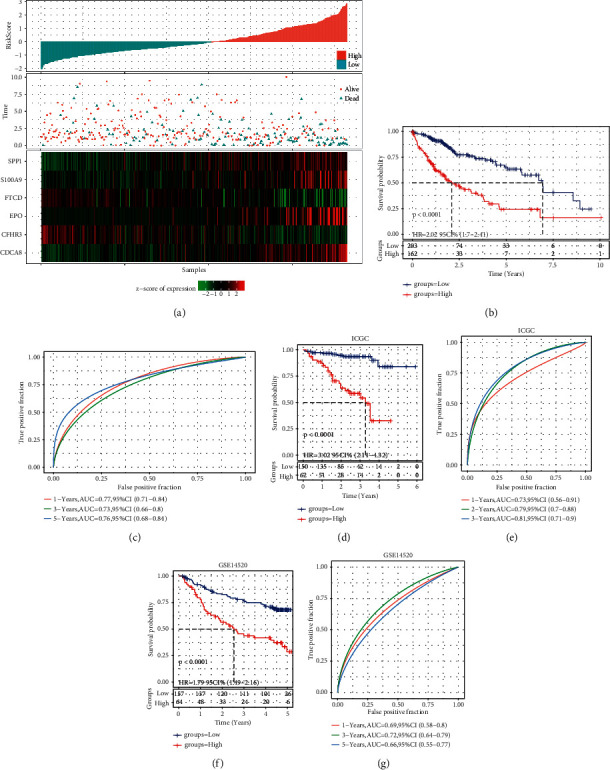
Generation and evaluation of risk scoring models based on six FRGs. (a) The risk plots of TCGA-LIHC showed the expression, survival status, and risk values distribution of the 6 FRGs of each HCC patient. (b) Kaplan–Meier curve for the OS of HCC patients in low- and high-risk groups in the TCGA-LIHC cohort. (c) ROC curves for the predictive significance of risk scores for OS at 1, 3, and 5 years in the TCGA-LIHC cohort. (d) Kaplan–Meier survival analysis between low- and high-risk patients in the ICGC cohort. (e) ROC curve was employed to examine predictive efficacy of the FPRS model over one, three, and five years in the ICGC cohort. (f) Kaplan–Meier curves of the FPRS model for HCC patients in various risk groups in the GSE14520 cohort. (g) Time-dependent ROC curves for the FPRS model in the GSE14520 cohort.

The expression and coefficient of 6 FRGs were used to construct the ferroptosis prognosis model, which was used to calculate the risk value of HCC samples and rank them. According to the cut-off point, 203 samples were classified into the low-FPRS group and 162 samples into the high-FPRS group. The risk plots of TCGA-LIHC illustrated the expression, survival status, and risk values distribution of the 6 FRGs of each HCC patient (Figure 6(a)). The Kaplan–Meier survival curve showed obvious differences in OS among TCGA-LIHC groups (Figure 6(b)). The area under the curve (AUC) for one-, three-, and five-year OS was 0.77, 0.732, and 0.76, respectively (Figure 6(c)). In ICGC and GSE14520 external validation sets, the survival advantage of low-risk samples was considerably greater as opposed to that of high-risk samples (Figures 6(d) and 6(f)). ROC curve showed that the FPRS model can effectively predict one-, three-, and five-year OS of HCC patients in the ICGC cohort and GSE14520 cohorts (Figures 6(e) and 6(g)). Furthermore, we also compared the expression distribution of six FRGs in two molecular subtypes. It can be observed that CDCA8, SPP1, S100A9, EPO, and FTCD are significantly overexpressed in C1 and CFHR3 is significantly overexpressed in C2 (Figure S2(a)). In addition, among the six FRGs, CDCA8, SPP1, S100A9, and EPO were significantly positively correlated with FPRS, and FTCD and CFHR3 were significantly negatively correlated with FPRS (Figure S2(b)). We used the string database to analyze the interaction between the six FRGs. It can be observed that there is no direct interaction between the six FRGs, suggesting that these genes may independently participate in different biological processes (Figure S2(c)).

### 3.7. The Manifestations of FPRS in Different Clinicopathological Features and Subtypes

**Figure 7:**
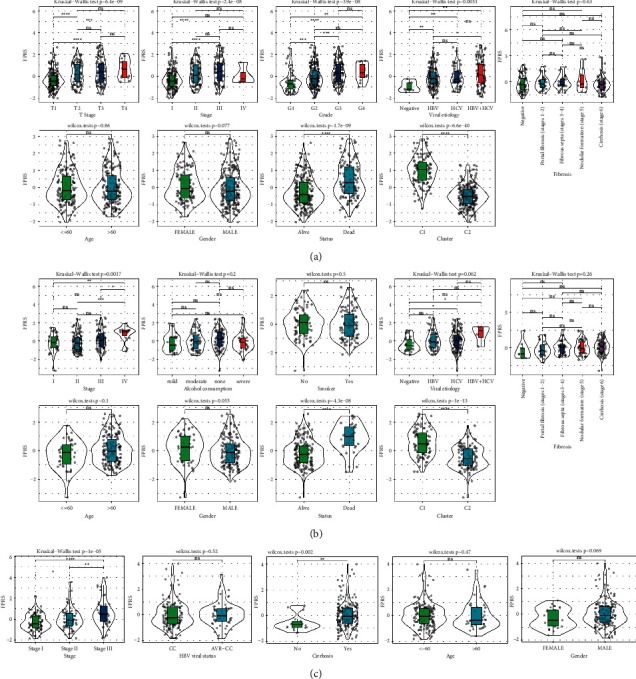
Association between FPRS and clinicopathological characteristics. (a) The violin plot showed the FPRS distributions according to age, gender, AJCC stage, grade, viral etiology, fibrosis, survival state, and molecular subtype in the ICGC cohort. (b) Correlation between FPRS and pathological features of samples in the GSE14520 cohort, including age, gender, AJCC stage, HBV viral status, and cirrhosis. Wilcoxon test was utilized for comparing the two groups, and the Kruskal-Wallis test was utilized for the differences between the two groups. ^*∗*^*P* < 0.05; ^*∗∗*^*P* < 0.01; ^*∗∗∗*^*P* < 0.001; ^*∗∗∗∗*^*P* < 0.0001. (c) The relationship between FPRS and pathological features of samples in the GSE76427 cohort.

When we studied the relationship between FPRS and clinical features, it was established that the FPRS was associated with pathological characteristics in TCGA-LIHC datasets, including T stage, AJCC stage, grade, viral etiology, and survival status. In addition, the distributions of FPRS were substantially varied between the two molecular subgroups (Figure 7(a)). We found that, in the ICGC cohort, FPRS was significantly correlated with the AJCC stage, life status, and molecular subtypes, but not with age, sex, smoking, viral etiology, and fibrosis of HCC patients (Figure 7(b)). In the GSE14520 cohort, FPRS was related to the AJCC stage and cirrhosis (Figure 7(c)).

### 3.8. Comparison of Molecular and Immune Characteristics Using FPRS

**Figure 8:**
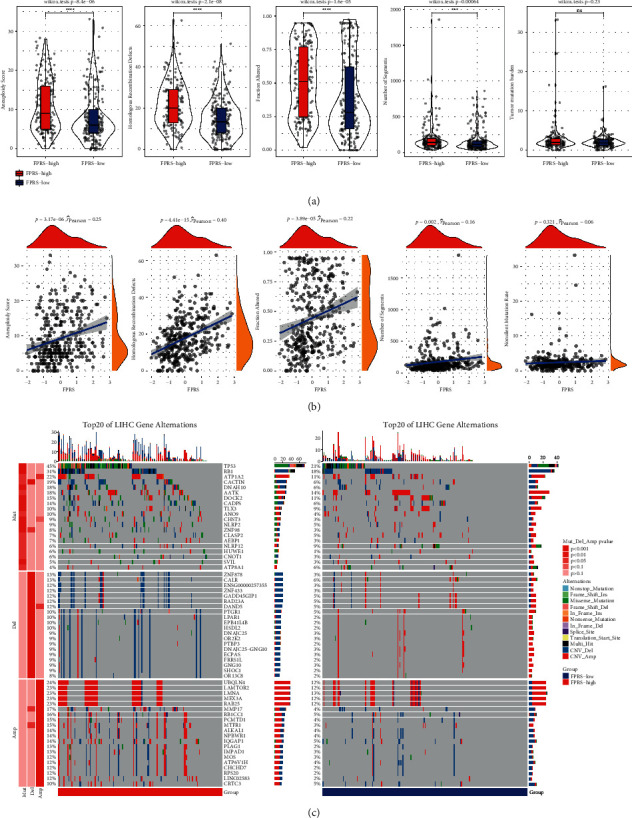
Molecular characteristics analysis between the high-FPRS and low-FPRS groups. (a) The difference of DNA damage-related index between high-FPRS and low-FPRS groups; Wilcoxon test. (b) Pearson's correlation analysis of FPRS and DNA damage-related indexes. (c) OncoPrint of somatic mutation and CNV distribution between low-FPRS and high-FPRS groups; Fisher's test; ^*∗∗∗*^*P* < 0.001; ^*∗∗∗∗*^*P* < 0.0001.

We identified the relationship between FPRS and genomic changes. We found that the two risk groups have significantly different performance on aneuploidy score, HRDs, fraction altered, and the number of segments. High-FPRS samples had significantly higher levels of these DNA damage-related variables (Figure 8(a)). Correlation analysis also illustrated that FPRS had a positive correlation with the score, HRDs, fraction altered, and the number of segments (Figure 8(b)). Furthermore, the overall somatic mutation rate, copy number amplification, and deletion in high-FPRS samples were greatly elevated as opposed to the ones in low-FPRS samples (Figure 8(c)).

**Figure 9:**
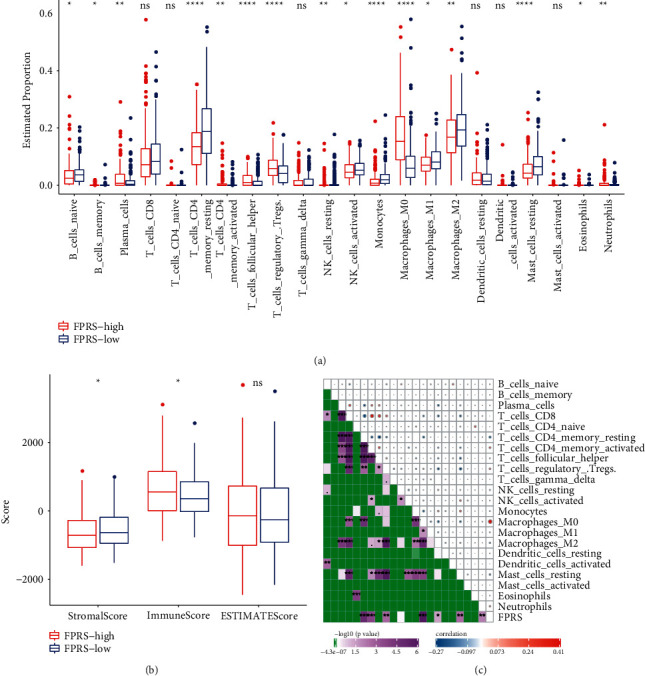
Immunological differences between different FPRS groups. (a) The estimated percentage of 22 different kinds of immune cells between different FPRS groups in the TCGA-LIHC cohort. (b) Difference of stromal, immune, and ESTIMATE scores between low-FPRS and high-FPRS groups. (c) Pearson's correlation analysis between 22 immune cells and FPRS.

To further study the immunological differences between distinct FPRS groups, the relative abundance of 22 different kinds of immune cells was computed utilizing CIBERSORT. The results showed that 16 kinds of immune cells showed significantly different estimated proportions in high-FPRS and low-FPRS groups and the proportion of immune cells enriched in the low-FPRS group was higher (Figure 9(a)). The stromal score of the low-FPRS group was greatly elevated in contrast to that of the high-FPRS group, while the immune score was greatly decreased than in high-FPRS group (Figure 9(b)). FPRS was also related to the levels of resting CD4 memory T cell, activated CD 4 memory T cell, neutrophils, regulated T cells, resting dendritic cells, and M0 macrophages (Figure 9(c)). FPRS was closely related to CNVs, DNA damage, and immune characteristics of HCC patients.

### 3.9. The Application of FPRS in Predicting Immune Chemotherapies

**Figure 10:**
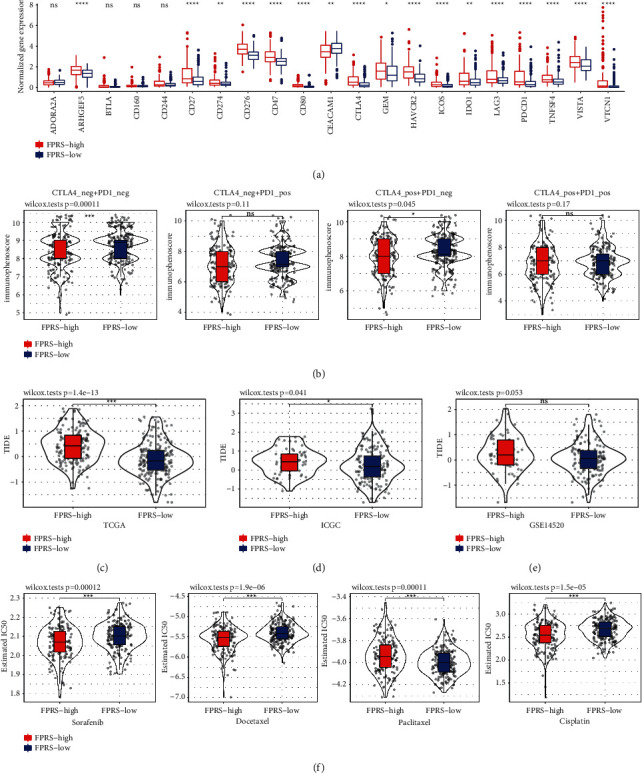
The role of the FPRS model in the prediction of immune/chemotherapeutic benefits. (a) Expression of 21 immune checkpoint-related genes in low-FPRS and high-FPRS patients. (b) The effect of different FPRS samples on IPS of anti-CTLA4 therapy, anti-PD1 therapy, and anti-CTLA4 and anti-PD1 combined therapy. (c) The violin chart illustrated the difference in TIDE scores between high FPRS and low FPRS in the TCGA-LIHC cohort. (d) In the ICGC cohort, the difference of TIDE score between low-FPRS and high-FPRS samples. (e) In the GSE14520 cohort, the performance of TIDE score on high FPRS and low FPRS. (f) Differential chemotherapeutic response between low-FPRS and high-FPRS groups based on IC50 available in the TCGA-LIHC database. Wilcoxon test; ^*∗*^*P* < 0.05; ^*∗∗*^*P* < 0.01; ^*∗∗∗*^*P* < 0.001; ^*∗∗∗∗*^*P* < 0.0001.

To determine whether FPRS can predict the response of HCC patients to immune checkpoint inhibitor (ICI) therapies, 21 immune checkpoint-related genes were obtained from HisgAtlas database [30] and their expression in high-FPRS and low-FPRS patients was analyzed. 17 immune checkpoint-related genes were found to have differential expression between low- and high-FPRS samples, and the expression level of 17 immune checkpoint-related genes in high-FPRS samples was greater in contrast with that in low-FPRS samples (Figure 10(a)). In addition, the applicability of different FPRS samples to anti-CTLA4 treatment, anti-PD1 treatment, anti-CTLA4, and anti-PD1 combined therapy was compared by IPS. The findings showed that the IPS of the low-FPRS group treated with anti-CTLA4 was relatively higher, indicating that the patients with low FPRS had a better therapeutic effect on anti-CTLA4 (Figure 10(b)). The high-FPRS patient had a greatly elevated TIDE score as opposed to that of the low-FPRS patient in the TCGA-LIHC cohort and ICGC cohort, indicating that a greater trend for immune escape was illustrated by the high-FPRS patient group, which may fail to respond to ICI treatment (Figures 10(c) and 10(d)). It is noteworthy that no significant differences were identified in the TIDE score between low-FPRS and high-FPRS groups in the GSE14520 cohort (Figure 10(e)). In addition, when evaluating the sensitivity of the two FPRS groups to sorafenib, docetaxel, paclitaxel, and cisplatin, we found that patients with high-FPRS had a greater sensitivity to sorafenib, docetaxel, and cisplatin, while patients with low FPRS had a greater sensitivity to paclitaxel (Figure 10(f)).

### 3.10. FPRS Combined with Clinicopathological Features of Nomogram Improves Prognosis and Survival Prediction

**Figure 11:**
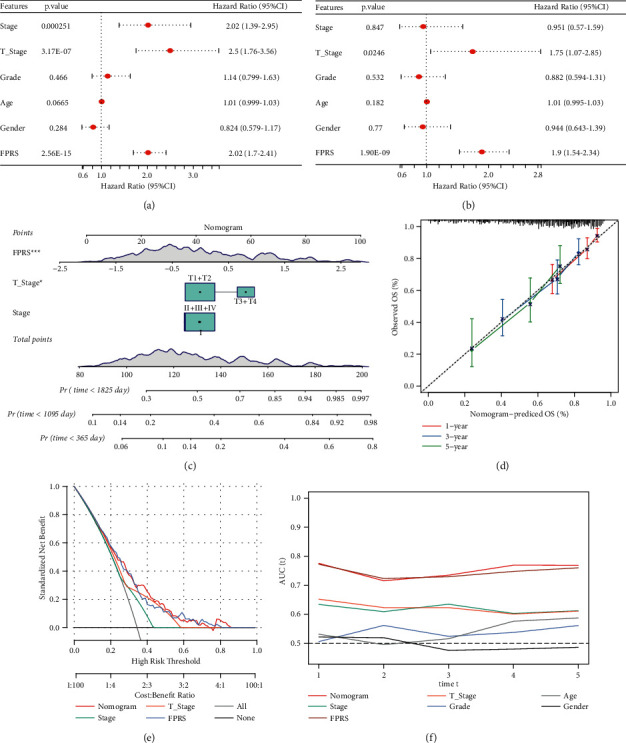
Nomogram of FPRS combined with clinicopathological features. (a) Univariate Cox regression analysis of the clinical variables. (b) Multivariate Cox regression analysis of the prognostic factors. (c) A nomogram based on FPRS and clinical factors was developed to predict the survival rate of HCC patients. Day counts were utilized to determine the survival time. (d) Calibration curve of the nomogram. (e) FPRS and clinical factors are shown in this decision curve analysis diagram. (f) AUC with different clinical variables predicting prognosis.

To construct a more effective nomogram model using the FPRS model and other clinicopathological information, multivariate and univariate Cox regression analysis showed that FPRS was an independent prognostic indicator of HCC (Figures 11(a) and11(b)). We established a nomogram including FPRS and several other clinical factors (AJCC stage and T stage) to anticipate OS of HCC patients and observed that FPRS made the greatest contribution to the survival prediction of nomogram (Figure 11(c)). The calibration curve illustrated that the anticipated probabilities of nomogram's one-, three-, and five-year OS were very close to the actually observed probabilities (Figure 11(d)). Decision curve analysis confirmed that the net income of FPRS and nomogram was considerably greater in contrast with that of the extreme curve and showed the strongest predictive ability of OS compared with other clinicopathological features (Figures 11(e) and 11(f)).

## 4. Discussion

Owing to the variability of HCC and the tumor microenvironment (TME) complexity, determining the long-term effectiveness of HCC continues to be a critical issue in clinical practice [33]. It is necessary to classify and refine the appropriate treatment intervention decision-making in this kind of tumor [34]. In addition, the effectiveness of sorafenib in treating advanced HCC strongly encourages the classification of HCC patients [34]. Several transcriptional group-based classifications were widely accepted in HCC [35–37] but lack genomic analysis. Recent studies have focused on defining different HCC categories based on more detailed biological characteristics to ensure maximum benefit and minimum toxicity for specific treatments [38]. Given the nonnegligible regulatory effect of sorafenib on ferroptosis, we revealed the molecular subclasses of HCC from the perspective of ferroptosis.

Transcriptome, genomic, and clinical data of 912 HCC samples were retrieved from TCGA, ICGC, and GEO. Based on the expression of 111 ferroptosis significantly associated with HCC prognosis, HCC samples from each cohort were separated into two heterogeneous subclasses, with significant differences in OS between the two subclasses. By comparing the clinical, genomic, and immune characteristics between the two subgroups, we recognized that, in C1 with poor prognosis, there were more patients with advanced stage and infection with HBV and HCV, higher rates of DNA damage including aneuploidy, HRD, fraction altered, and the number of segments, and higher probability of gene mutation and copy number variation. To some extent, these results reveal the reason for the poor prognosis of C1, because the TME cell components of HCC are mainly composed of HCC cells, HCC-related fibroblasts, endothelial cells, and immune cells. The TME cell components of HCC are mainly composed of HCC cells, HCC-related fibroblasts, immune cells, and endothelial cells [33]. Among them, immune cells are most often studied, because the infiltration levels of immune cells can largely reflect the applicability of patients to immunotherapy [39]. HCC patients with C1 had higher levels of M0 macrophages, regulatory T cells, helper T cells, and activated memory CD4 T cells infiltration and higher immune score. In C2, there is strong infiltration of resting memory CD4 T cells, naive B cells, monocyte, resting mast cells, and M1and M2 macrophages. Therefore, there was strong heterogeneity between C1 and C2, including clinical, molecular, and immunological features.

Additionally, we developed and validated a prognostic model called FPRS, which is composed of CDCA8, SPP1, EPO, S200A9, FTCD, and CFHR3 in three independent cohorts. It shows considerable effect in predicting the OS probability of HCC samples and can reflect the clinical characteristics of different patients. It is an independent prognostic factor for HCC. FPRS model assigned each sample with a specific risk score, and patients were subdivided into different risk groups according to such score. In line with our expectations, the prognosis of high FPRS was considerably unfavorable in contrast with that of low FPRS. Notably, from the study of Teresa Davoli, we learned that copy number aberration contributed more to immune characteristics than tumor mutation load and the low burden of copy number increase/loss is related to the responsiveness to immunotherapy [40]. Indeed, our results also found that the overall somatic mutation rate, copy number amplification, and deletion in low-FPRS samples were significantly lower than those in high-FPRS samples and low-FPRS samples were more effective in anti-CTLA4 therapy at immune checkpoints. Moreover, we predicted that patients who have low FPRS had a greater sensitivity to paclitaxel, while patients who have high FPRS had a greater sensitivity to sorafenib, docetaxel, and cisplatin.

In summary, on the one hand, our study revealed two ferroptosis subclasses, which showed heterogeneity in prognosis, clinical characteristics, genetic events, and immune characteristics. On the other hand, a classifier called the FPRS model has been developed and validated, which may help predict the prognosis and select patients suitable for immunotherapy.

## Data Availability

The data used to support the research are included within this manuscript.

## Supplementary Materials

Supplementary Materials Figure S1: work flow chart. Figure S2: expression relationship of 6 FRGs. A: the expression and distribution of 6 genes in two molecular subtypes were different; B: correlation between 6 gene expressions and FPRs; C: protein interaction network among 6 genes. Supplementary Table 1: list of 38 FRGs. Supplementary Table 2: molecular subtypes of each sample in TCGA dataset.
