# Supplementary material for: The Detection and Verification of Two Heterogeneous Subgroups and a Risk Model Based on Ferroptosis-Related Genes in Hepatocellular Carcinoma
Source: J Oncol. 2022 Mar 12;2022:1182383. doi: 10.1155/2022/1182383 (PMC8934225; doi:10.1155/2022/1182383)
Supplement: Supplementary Materials — Figure S1: work flow chart. Figure S2: expression relationship of 6 FRGs. A: the expression and distribution of 6 genes in two molecular subtypes were different; B: correlation between 6 gene expressions and FPRs; C: protein interaction network among 6 genes. Supplementary Table 1: list of 38 FRGs. Supplementary Table 2: molecular subtypes of each sample in TCGA dataset. [file 1182383.f1.zip › 1182383.f1/Supplementary Table1.pdf]

Supplementary Table1: List of 38 FRGs

FRGs
ALB
ARRDC3
ASNS
ATF4
AURKA
CAPG
CBS
DDIT3
DRD5
EIF2S1
ELAVL1
FTH1
FTL
GPT2
HMOX1
IL33
MAFG
MT3
NCF2
OXSR1
PCK2
PRDX1
RELA
RPL8
RRM2
SLC1A4
SLC2A1
SLC2A6
SLC3A2
SLC7A11
SRXN1
STMN1
TFRC
TRIB3
TXNIP
TXNRD1
VEGFA
ZFP69B
