# Supplementary material for: The Detection and Verification of Two Heterogeneous Subgroups and a Risk Model Based on Ferroptosis-Related Genes in Hepatocellular Carcinoma
Source: J Oncol. 2022 Mar 12;2022:1182383. doi: 10.1155/2022/1182383 (PMC8934225; doi:10.1155/2022/1182383)
Supplement: Supplementary Materials — Figure S1: work flow chart. Figure S2: expression relationship of 6 FRGs. A: the expression and distribution of 6 genes in two molecular subtypes were different; B: correlation between 6 gene expressions and FPRs; C: protein interaction network among 6 genes. Supplementary Table 1: list of 38 FRGs. Supplementary Table 2: molecular subtypes of each sample in TCGA dataset. [file 1182383.f1.zip › 1182383.f1/Supplementary Table2.pdf]

Supplementary Table2: Molecular subtypes of each sample in TCGA dataset

Sample	Cluster
TCGA-DD-A4NG-01A	C1
TCGA-G3-AAV4-01A	C2
TCGA-2Y-A9H1-01A	C2
TCGA-BC-A10Y-01A	C2
TCGA-K7-AAU7-01A	C1
TCGA-BC-A10W-01A	C1
TCGA-DD-AACV-01A	C2
TCGA-DD-AAD3-01A	C2
TCGA-DD-A1EI-01A	C1
TCGA-DD-AAC9-01A	C2
TCGA-DD-AACT-01A	C2
TCGA-GJ-A6C0-01A	C1
TCGA-CC-5258-01A	C1
TCGA-DD-AADP-01A	C2
TCGA-DD-AACW-01A	C2
TCGA-ZS-A9CD-01A	C2
TCGA-UB-A7MF-01A	C1
TCGA-WX-AA46-01A	C2
TCGA-CC-A8HV-01A	C1
TCGA-DD-AADQ-01A	C2
TCGA-DD-AADB-01A	C1
TCGA-DD-A1EA-01A	C2
TCGA-5R-AA1C-01A	C2
TCGA-DD-A113-01A	C2
TCGA-2Y-A9H9-01A	C2
TCGA-CC-A7IE-01A	C1
TCGA-XR-A8TD-01A	C1
TCGA-DD-AAD2-01A	C2
TCGA-DD-AACY-01A	C2
TCGA-CC-A7IF-01A	C2
TCGA-DD-AACD-01A	C2
TCGA-ED-A4XI-01A	C2
TCGA-CC-A9FW-01A	C1
TCGA-G3-A5SJ-01A	C1
TCGA-DD-AAE2-01A	C2
TCGA-G3-A7M9-01A	C1
TCGA-EP-A2KC-01A	C2
TCGA-2Y-A9GS-01A	C1
TCGA-ED-A7PX-01A	C1
TCGA-DD-AADC-01A	C1
TCGA-DD-AAVZ-01A	C2

TCGA-CC-A3MA-01A	C1
TCGA-2Y-A9GY-01A	C1
TCGA-ED-A627-01A	C2
TCGA-2Y-A9GW-01A	C1
TCGA-DD-AADO-01A	C1
TCGA-DD-AADK-01A	C2
TCGA-DD-A1EH-01A	C2
TCGA-G3-AAV7-01A	C1
TCGA-DD-AACA-01A	C2
TCGA-FV-A3R3-01A	C2
TCGA-KR-A7K0-01A	C2
TCGA-DD-AACC-01A	C1
TCGA-BC-A3KG-01A	C2
TCGA-G3-AAV2-01A	C2
TCGA-CC-A7IK-01A	C2
TCGA-G3-A25S-01A	C1
TCGA-DD-AADR-01A	C2
TCGA-UB-A7MD-01A	C1
TCGA-DD-A4NS-01A	C2
TCGA-DD-AAE3-01A	C2
TCGA-DD-AACP-01A	C1
TCGA-2Y-A9H8-01A	C2
TCGA-FV-A3I1-01A	C1
TCGA-2Y-A9H7-01A	C2
TCGA-DD-AAVU-01A	C1
TCGA-BC-A110-01A	C2
TCGA-ZP-A9CZ-01A	C2
TCGA-DD-AAE1-01A	C2
TCGA-GJ-A3OU-01A	C1
TCGA-RC-A7SB-01A	C2
TCGA-DD-AAVS-01A	C2
TCGA-CC-A8HS-01A	C1
TCGA-FV-A3I0-01A	C1
TCGA-2Y-A9GT-01A	C2
TCGA-2Y-A9HA-01A	C1
TCGA-FV-A2QQ-01A	C2
TCGA-DD-AAEH-01A	C2
TCGA-DD-AADM-01A	C2
TCGA-G3-A3CK-01A	C2
TCGA-EP-A26S-01A	C2
TCGA-DD-A11A-01A	C2
TCGA-DD-AAE6-01A	C2
TCGA-XR-A8TE-01A	C2
TCGA-DD-A73B-01A	C2

TCGA-EP-A3JL-01A	C1
TCGA-CC-A3MB-01A	C2
TCGA-DD-AAD0-01A	C2
TCGA-MI-A75I-01A	C1
TCGA-DD-AACZ-01A	C1
TCGA-CC-A8HU-01A	C1
TCGA-RC-A6M5-01A	C2
TCGA-2Y-A9GZ-01A	C2
TCGA-DD-AACO-01A	C2
TCGA-LG-A6GG-01A	C2
TCGA-BC-4073-01B	C1
TCGA-ED-A7PZ-01A	C1
TCGA-DD-A4NV-01A	C2
TCGA-DD-A73G-01A	C2
TCGA-G3-AAV3-01A	C1
TCGA-G3-A25U-01A	C2
TCGA-BC-A5W4-01A	C2
TCGA-UB-AA0U-01A	C2
TCGA-EP-A12J-01A	C2
TCGA-G3-A5SI-01A	C2
TCGA-FV-A4ZQ-01A	C1
TCGA-DD-A4ND-01A	C2
TCGA-ED-A8O5-01A	C2
TCGA-UB-A7ME-01A	C2
TCGA-CC-5264-01A	C1
TCGA-DD-AADA-01A	C2
TCGA-2Y-A9HB-01A	C2
TCGA-DD-AACS-01A	C2
TCGA-DD-AAE0-01A	C1
TCGA-G3-A5SK-01A	C2
TCGA-BC-A69I-01A	C2
TCGA-BC-A10R-01A	C2
TCGA-BC-A10X-01A	C2
TCGA-BC-A10S-01A	C2
TCGA-DD-AAVX-01A	C2
TCGA-DD-AAVQ-01A	C2
TCGA-RC-A7SH-01A	C2
TCGA-G3-A3CJ-01A	C2
TCGA-DD-A115-01A	C2
TCGA-BC-A10Q-01A	C1
TCGA-DD-A1EE-01A	C1
TCGA-ZP-A9CY-01A	C2
TCGA-FV-A23B-01A	C2
TCGA-HP-A5MZ-01A	C2

TCGA-DD-AACK-01A	C2
TCGA-CC-A1HT-01A	C1
TCGA-DD-A11B-01A	C2
TCGA-ZP-A9D4-01A	C2
TCGA-HP-A5N0-01A	C2
TCGA-EP-A2KB-01A	C2
TCGA-DD-A4NN-01A	C1
TCGA-ES-A2HS-01A	C2
TCGA-G3-A3CG-01A	C2
TCGA-DD-AAVR-01A	C2
TCGA-DD-AAD1-01A	C1
TCGA-DD-A73D-01A	C2
TCGA-DD-AAVW-01A	C2
TCGA-DD-A4NF-01A	C2
TCGA-DD-AACN-01A	C2
TCGA-DD-A3A2-01A	C2
TCGA-K7-A5RF-01A	C2
TCGA-DD-A116-01A	C1
TCGA-DD-A4NI-01A	C2
TCGA-BC-A10T-01A	C2
TCGA-DD-AADJ-01A	C2
TCGA-DD-AACE-01A	C2
TCGA-FV-A3R2-01A	C1
TCGA-DD-AAE7-01A	C2
TCGA-DD-AADD-01A	C2
TCGA-4R-AA8I-01A	C2
TCGA-FV-A496-01A	C2
TCGA-DD-A39X-01A	C1
TCGA-DD-AADS-01A	C2
TCGA-DD-AAE9-01A	C2
TCGA-G3-AAUZ-01A	C2
TCGA-WX-AA47-01A	C2
TCGA-NI-A4U2-01A	C2
TCGA-DD-AACI-01A	C2
TCGA-ZP-A9D1-01A	C2
TCGA-UB-A7MB-01A	C2
TCGA-DD-AAVP-01A	C2
TCGA-DD-A4NR-01A	C1
TCGA-RG-A7D4-01A	C1
TCGA-5R-AAAM-01A	C2
TCGA-DD-A3A4-01A	C2
TCGA-BC-A217-01A	C2
TCGA-DD-A4NQ-01A	C1
TCGA-UB-A7MC-01A	C2

TCGA-MR-A8JO-01A	C1
TCGA-DD-AAEG-01A	C2
TCGA-DD-A39W-01A	C2
TCGA-DD-A39Z-01A	C2
TCGA-BC-A10Z-01A	C2
TCGA-WQ-AB4B-01A	C2
TCGA-CC-A123-01A	C1
TCGA-YA-A8S7-01A	C1
TCGA-2Y-A9H2-01A	C1
TCGA-5C-A9VG-01A	C2
TCGA-CC-A3M9-01A	C1
TCGA-EP-A2KA-01A	C2
TCGA-DD-AADW-01A	C1
TCGA-DD-AAED-01A	C2
TCGA-LG-A9QD-01A	C2
TCGA-DD-AADV-01A	C2
TCGA-G3-A3CH-01A	C2
TCGA-G3-AAV1-01A	C2
TCGA-ZS-A9CG-01A	C2
TCGA-DD-AAW3-01A	C2
TCGA-ED-A7PY-01A	C2
TCGA-ZP-A9D2-01A	C1
TCGA-G3-A25Y-01A	C1
TCGA-G3-A3CI-01A	C2
TCGA-K7-A5RG-01A	C1
TCGA-EP-A3RK-01A	C1
TCGA-CC-A7IJ-01A	C1
TCGA-MI-A75H-01A	C2
TCGA-G3-AAV5-01A	C2
TCGA-ED-A7XP-01A	C1
TCGA-DD-A1ED-01A	C2
TCGA-ED-A5KG-01A	C1
TCGA-ED-A459-01A	C1
TCGA-2Y-A9GX-01A	C2
TCGA-DD-A73E-01A	C2
TCGA-QA-A7B7-01A	C2
TCGA-DD-AACL-01A	C1
TCGA-MR-A520-01A	C2
TCGA-DD-A1EF-01A	C1
TCGA-G3-A25T-01A	C1
TCGA-FV-A4ZP-01A	C1
TCGA-WX-AA44-01A	C1
TCGA-DD-A4NE-01A	C2
TCGA-CC-A7IG-01A	C1

TCGA-ZP-A9D0-01A	C2
TCGA-G3-A25X-01A	C1
TCGA-BC-4072-01B	C1
TCGA-XR-A8TC-01A	C2
TCGA-BC-A3KF-01A	C2
TCGA-O8-A75V-01A	C2
TCGA-DD-AADI-01A	C2
TCGA-BC-A8YO-01A	C1
TCGA-DD-A39V-01A	C2
TCGA-DD-A1EC-01A	C1
TCGA-DD-AACQ-01A	C2
TCGA-DD-A1EB-01A	C2
TCGA-KR-A7K7-01A	C1
TCGA-DD-AAVV-01A	C1
TCGA-DD-AADN-01A	C1
TCGA-LG-A9QC-01A	C2
TCGA-RC-A7S9-01A	C2
TCGA-BC-A112-01A	C1
TCGA-2Y-A9H0-01A	C1
TCGA-CC-A7IL-01A	C2
TCGA-KR-A7K2-01A	C2
TCGA-DD-AADU-01A	C2
TCGA-ED-A66Y-01A	C2
TCGA-CC-A8HT-01A	C1
TCGA-DD-AADY-01A	C2
TCGA-BC-A216-01A	C1
TCGA-RC-A7SF-01A	C2
TCGA-RC-A7SK-01A	C2
TCGA-DD-AAD8-01A	C2
TCGA-DD-AAEE-01A	C2
TCGA-DD-A119-01A	C2
TCGA-G3-AAV6-01A	C1
TCGA-DD-A73F-01A	C2
TCGA-DD-A4NP-01A	C2
TCGA-DD-AAEK-01A	C2
TCGA-DD-A11D-01A	C2
TCGA-DD-A1EG-01A	C1
TCGA-PD-A5DF-01A	C1
TCGA-DD-A3A9-01A	C2
TCGA-2Y-A9H4-01A	C2
TCGA-DD-A118-01A	C2
TCGA-DD-A4NL-01A	C2
TCGA-5C-A9VH-01A	C2
TCGA-3K-AAZ8-01A	C2

TCGA-MI-A75G-01A	C2
TCGA-DD-A3A6-01A	C1
TCGA-CC-A5UD-01A	C1
TCGA-DD-A3A3-01A	C2
TCGA-CC-A7IH-01A	C2
TCGA-DD-A4NH-01A	C1
TCGA-K7-A6G5-01A	C2
TCGA-DD-AACU-01A	C2
TCGA-BW-A5NO-01A	C2
TCGA-XR-A8TG-01A	C2
TCGA-DD-A1EJ-01A	C1
TCGA-CC-5259-01A	C2
TCGA-2Y-A9H3-01A	C1
TCGA-UB-AA0V-01A	C2
TCGA-GJ-A9DB-01A	C2
TCGA-DD-A4NK-01A	C2
TCGA-2Y-A9H6-01A	C2
TCGA-BD-A2L6-01A	C2
TCGA-G3-A5SM-01A	C2
TCGA-DD-AAD5-01A	C1
TCGA-BD-A3EP-01A	C1
TCGA-CC-5260-01A	C1
TCGA-WQ-A9G7-01A	C1
TCGA-FV-A2QR-01A	C2
TCGA-DD-AAW2-01A	C2
TCGA-DD-AA3A-01A	C1
TCGA-CC-5263-01A	C1
TCGA-FV-A495-01A	C2
TCGA-BC-A10U-01A	C1
TCGA-ZS-A9CE-01A	C2
TCGA-G3-A7M8-01A	C2
TCGA-5R-AA1D-01A	C2
TCGA-ED-A82E-01A	C1
TCGA-DD-A3A1-01A	C1
TCGA-MI-A75E-01A	C2
TCGA-DD-AAEA-01A	C2
TCGA-ZS-A9CF-01A	C2
TCGA-G3-A25V-01A	C2
TCGA-CC-A9FS-01A	C2
TCGA-BC-A69H-01A	C1
TCGA-DD-A3A7-01A	C1
TCGA-CC-5261-01A	C1
TCGA-G3-AAV0-01A	C2
TCGA-DD-AAE4-01A	C2

TCGA-CC-A7II-01A	C1
TCGA-DD-A3A8-01A	C2
TCGA-G3-A7M5-01A	C2
TCGA-CC-A3MC-01A	C1
TCGA-ED-A66X-01A	C1
TCGA-G3-A6UC-01A	C2
TCGA-BD-A3ER-01A	C2
TCGA-DD-A3A5-01A	C2
TCGA-DD-AACH-01A	C1
TCGA-CC-5262-01A	C1
TCGA-DD-AADG-01A	C2
TCGA-DD-AAW0-01A	C2
TCGA-DD-AACJ-01A	C2
TCGA-DD-AADL-01A	C2
TCGA-RC-A6M6-01A	C1
TCGA-DD-A4NO-01A	C2
TCGA-CC-A5UE-01A	C1
TCGA-G3-A7M7-01A	C2
TCGA-ES-A2HT-01A	C2
TCGA-DD-AACX-01A	C2
TCGA-2Y-A9GV-01A	C2
TCGA-DD-AACF-01A	C2
TCGA-UB-A7MA-01A	C1
TCGA-G3-A25Z-01A	C2
TCGA-MI-A75C-01A	C2
TCGA-DD-A4NA-01A	C1
TCGA-ZP-A9CV-01A	C2
TCGA-T1-A6J8-01A	C2
TCGA-DD-A1EK-01A	C2
TCGA-5C-AAPD-01A	C1
TCGA-KR-A7K8-01A	C1
TCGA-DD-AAD6-01A	C2
TCGA-DD-A4NB-01A	C2
TCGA-DD-A11C-01A	C2
TCGA-WJ-A86L-01A	C2
TCGA-ED-A8O6-01A	C1
TCGA-DD-AAEB-01A	C2
TCGA-2Y-A9GU-01A	C2
TCGA-DD-A73A-01A	C2
TCGA-RC-A6M4-01A	C2
TCGA-DD-AAVY-01A	C2
TCGA-DD-A1EL-01A	C1
TCGA-DD-A4NJ-01A	C2
TCGA-G3-A5SL-01A	C2

TCGA-DD-AACG-01A	C1
TCGA-DD-AAC8-01A	C1
TCGA-ED-A7XO-01A	C2
TCGA-DD-AACB-01A	C1
TCGA-G3-A7M6-01A	C1
TCGA-ED-A97K-01A	C1
TCGA-DD-A73C-01A	C2
TCGA-DD-AAW1-01A	C2
TCGA-XR-A8TF-01A	C2
TCGA-CC-A5UC-01A	C1
TCGA-DD-A39Y-01A	C1
TCGA-DD-A114-01A	C1
TCGA-NI-A8LF-01A	C2
TCGA-DD-AADF-01A	C2
TCGA-DD-AAEI-01A	C2
TCGA-2Y-A9H5-01A	C2
