# Supplementary figures and images for: The Detection and Verification of Two Heterogeneous Subgroups and a Risk Model Based on Ferroptosis-Related Genes in Hepatocellular Carcinoma
Source: J Oncol. 2022 Mar 12;2022:1182383. doi: 10.1155/2022/1182383 (PMC8934225; doi:10.1155/2022/1182383)

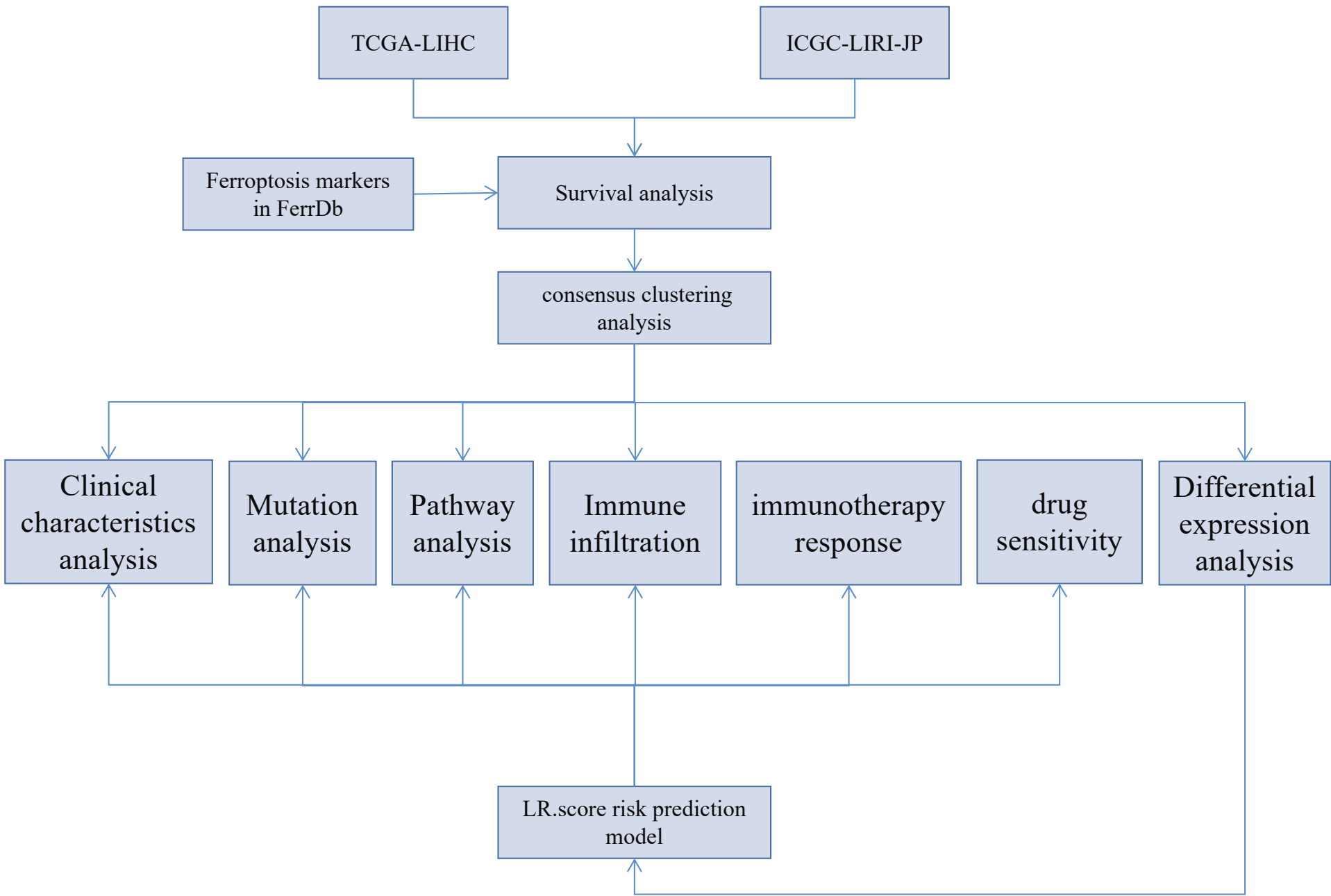

Supplement: Supplementary Materials — Figure S1: work flow chart. Figure S2: expression relationship of 6 FRGs. A: the expression and distribution of 6 genes in two molecular subtypes were different; B: correlation between 6 gene expressions and FPRs; C: protein interaction network among 6 genes. Supplementary Table 1: list of 38 FRGs. Supplementary Table 2: molecular subtypes of each sample in TCGA dataset. [file 1182383.f1.zip › 1182383.f1/Figure S1.pdf]
