# Supplementary material for: The Detection and Verification of Two Heterogeneous Subgroups and a Risk Model Based on Ferroptosis-Related Genes in Hepatocellular Carcinoma
Source: J Oncol. 2022 Mar 12;2022:1182383. doi: 10.1155/2022/1182383 (PMC8934225; doi:10.1155/2022/1182383)
Supplement: Supplementary Materials — Figure S1: work flow chart. Figure S2: expression relationship of 6 FRGs. A: the expression and distribution of 6 genes in two molecular subtypes were different; B: correlation between 6 gene expressions and FPRs; C: protein interaction network among 6 genes. Supplementary Table 1: list of 38 FRGs. Supplementary Table 2: molecular subtypes of each sample in TCGA dataset. [file Figure S2.pdf › 1182383.f1/Figure S2.pdf]

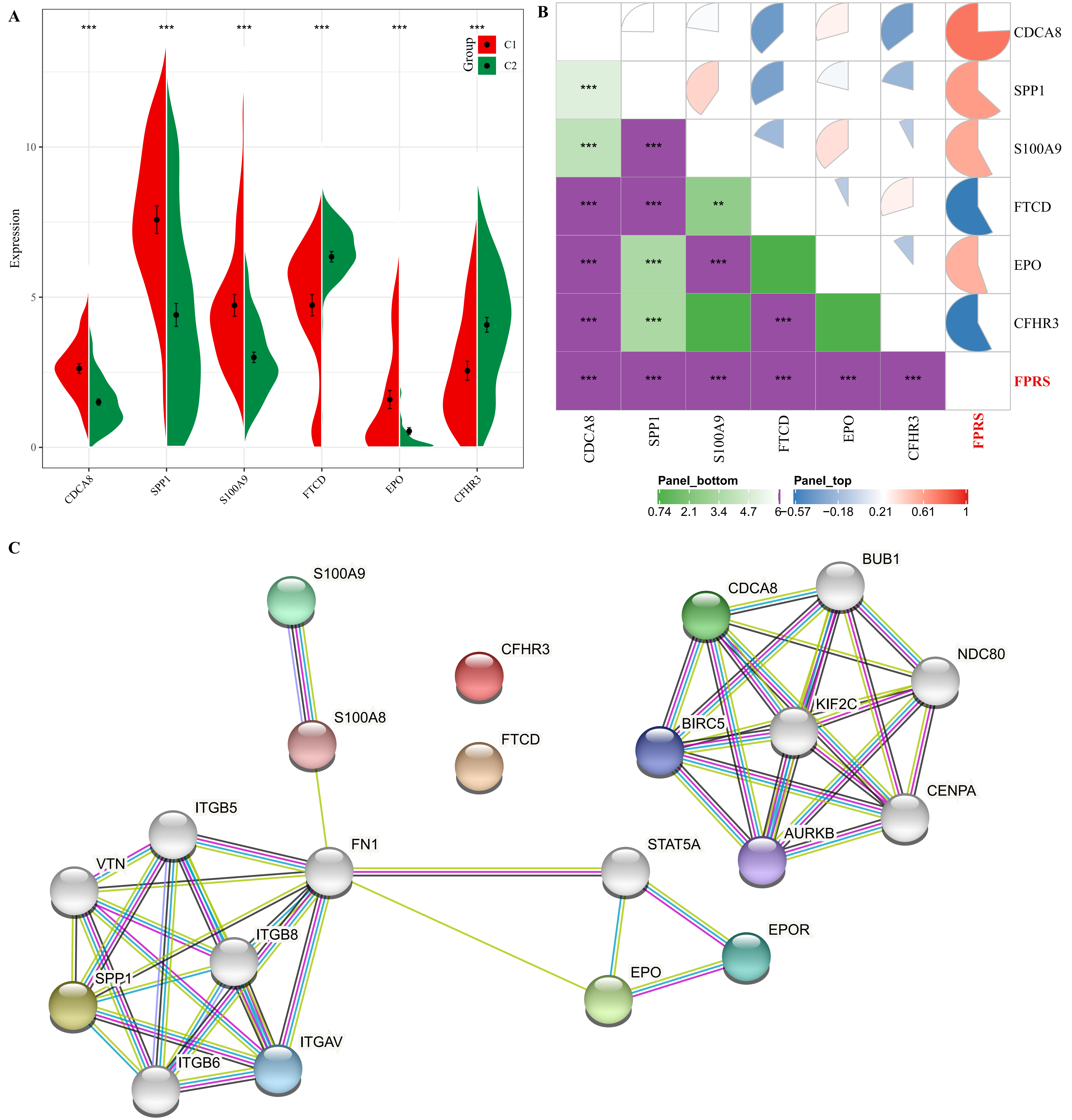
A
***
***
***
***
***
***
Group
C1
C2
Expression
10
5
0
CDCA8
SPP1
S100A9
FTCD
EPO
CFHR3
B
CDCA8
SPP1
S100A9
FTCD
EPO
CFHR3
FPRS
***
**
Panel_bottom
0.74 2.1 3.4 4.7 6
Panel_top
−0.57 −0.18 0.21 0.61 1
C
S100A9
S100A8
CFHR3
FTCD
BUB1
CDCA8
NDC80
KIF2C
BIRC5
CENPA
AURKB
ITGB5
VTN
FN1
STAT5A
ITGB8
SPP1
EPOR
EPO
ITGAV
ITGB6
